# Obstetrics

**Published:** 1876-07

**Authors:** 


### III. Obstetrics.

1. *On Laceration of the Gravid Uterus.* J. Ashburton Thompson. (*Obs. Jour. of Great Britain*, Jan., Feb. and March, 1876.)

The majority of obstetric writers agree upon the following seven symptoms as being characteristic of laceration of the gravid uterus; viz.: 1. Violence of the throes before rupture. 2. A peculiar pain at the time of rupture. 3. Hæmorrhage. 4. Immediate cessation of the throes. 5. Retrocession of the presentation. 6. The speedy occurrence of collapse. 7. Convulsions.

Most writers agree further, in omitting a consideration of exceptional cases of rupture; thus allowing the inference that the typical case of rupture occurs the most frequently. This paper is an examination of twenty-three cases of laceration of the gravid uterus; not selected cases, but cases collected from journals and Societies' transactions, between the years 1857 and 1873. In the paper this series of collected cases is examined with reference to the occurrence of the typical symptoms in the order which has been given :

*Of the Character of the Throes preceding Rupture.*—In eleven of Thompson's cases, the throes appear to have been of the usual kind. Of seven others it was uncertain whether there was a regular contraction of the uterus at any time. In four other cases, the laceration was traumatic, occurring under operation. In five other cases, the throes were variously modified. "Thus, omitting the seven cases already excepted, in five out of sixteen the throes were modified before rupture occurred; but from these five the last must be subtracted, if this symptom be regarded as a prognostic, for it was only the throe attended by rupture which was at all peculiar. 26.6 is the percentage, then, in which the throes were peculiarly aggravated before rupture in this series of cases."

*Of Pain occurring at the time of Rupture.*—The pain caused by the tearing of the uterine substance has been described as sudden, and excruciatingly sharp. Should it occur at the moment of contraction of the organ, this pain constitutes an element which is clearly distinguishable from the ordinary throe. This pain did not occur in the four cases of traumatic rupture ; excluding these cases, in eleven of the nineteen remaining cases, this peculiar pain was not observed. In eight cases this tearing pain was noted, but the word "excruciating" seldom occurs in these clinical reports. In about 58.5 per cent., then, of the cases of laceration, a peculiar pain was not observed.

*Of Hæmorrhage.*—This may be either external or internal. External hæmorrhage is a rare consequence of rupture. It is spoken of but once in this series ; in this instance it occurred before labor set in, and might have been of the variety known as accidental hæmorrhage. Internal hæmorrhage is more common, but it can be of but little utility as a diagnostic.

In a very interesting case of Dr. Jolly, a rapidly growing tumor was observed within the abdomen during labor; the bladder being empty, Dr. Jolly was led to surmise that a rupture had taken place, and that the tumor was a collection of blood in the peritoneal cavity. This proved to be the case. As a rule, the effusion is too small to be discovered before death. On the whole, then, hæmorrhage occurs but rarely; though it is reasonable to suppose that hæmorrhage would be inevitable if the vaginal portion of the uterine cervix, or the vagina itself, be also involved in the rent.

*Of the Action of the Uterus subsequent to Rupture.*—The greatest stress has always been laid on the sudden cessation of uterine action as a diagnostic of rupture; and but two writers, (Leishman and Jolly) have written of exceptions to the rule. Of the present series of cases, four, the traumatic cases, must be omitted. In two of the remaining cases the point is not noted; this leaves a remainder of seventeen cases. Of these, in nine cases the throes ceased suddenly upon rupture. In eight, the throes were either modified in some manner, or they remained unaltered. In but 52.9 per cent. of the cases, then, a little more than one-half, the throes ceased in a characteristic manner.

*Of Retrocession of the Presentation*—At most, this can only be regarded as confirmatory. The presenting part of the fœtus cannot recede provided the head is impacted in the excavation; nor will the body of the fœtus recede if the head is in the world. Further, if the uterus should contract spasmodically after the rupture, the fœtus will not escape into the abdominal cavity, even though the rent be large enough to admit of its passage. If this series is analyzed with reference to this symptom, it will be found that the fœtus receded in four instances. In six other cases retrocession was not noted. In two cases the fœtus did not recede, the head being impacted. In one case, the expulsion of the head coincided with the rupture, no retrocession. And in three other cases there was no retrocession of the presentation, though the rent was large. In four cases the rupture occurred during operation.

*Of the speedy occurrence of Collapse.*—This symptom has been thought as characteristic of rupture as the sudden cessation of the throes. In three of these cases, the time when the collapse occurred is not noted. It is doubtful in another. In another the patient was under the influence of chloroform, and collapse was not observed; for this reason this case is omitted, though it might fairly be included because another case is included under similar circumstances, when collapse did occur, and was thought to be due to the anæsthetic. In the eighteen remaining cases, collapse occurred suddenly upon the completion of the rupture in six instances. In six other cases, it did not occur till hours or minutes after rupture. Collapse did not occur till hours or minutes after labor in six other cases. In but six of the eighteen cases, in but 33.33 per cent., did collapse suddenly supervene upon the rupture. In one case, the patient was standing upright when first seen, and rupture of the uterus was not suspected for some time afterward. In this series of cases, the degree of collapse bears no sort of relation to the extent of injury which the uterus has suffered. * * * "The evidence of shock manifested in any particular case will depend upon other circumstances than the size of the wound, or, probably, the mode in which it occurs. It may be that the chief of these circumstances is the vital energy of the individual, which varies in each person ; and with it the rate at which collapse appears may be expected to vary too. Cases are on record in which the fact of laceration has been discovered accidentally during a vaginal examination; no symptoms of any kind attending or ensuing upon it."

*Convulsions.*—In this series of cases convulsions have not once been noted.

From an examination of this series of cases of Thompson's, it is evident that the description in the books of a typical case of laceration of the gravid uterus cannot be applied to the majority of cases of this injury. In other words, the so-called typical case occurs but rarely, while the cases that are but barely hinted at by a few writers as exceptional, do occur more frequently than the former variety.

The medico-legal application of the foregoing facts, which is the author's real object, is of the greatest importance to practitioners of obstetrics. "In order to establish the culpability of the attendant, one has a right to demand that it should be shown, first of all, that no predisposing cause of rupture existed ; and only in the second place will it be necessary to show that the determining cause was some other than the contraction of the uterus itself." The remote predisposing causes are previous laceration, Cæsarian section, and peri-uterine inflammations. The latter especially, changing the integrity of the uterine walls, rendering them liable to give way under the most trivial exciting causes. Thus in one case, the sudden contraction of the abdominal muscles, which the subject exercised to save herself from falling, was sufficient to rupture the uterus. In a case of Dr. Barnes, the uterus was lacerated spontaneously ; there being apparently no change in the uterine substance. To exclude all predisposing causes in a given case, the strictest inquiry into all the antecedents of the patient is essential, especially with reference to any previous pelvic inflammation.

In the second place, before it can be said that any operative interference on the part of the attendant caused the rupture, it should be established that a rupture did not exist before the interference was made. This is not always easy, and requires the closest examination of all the symptoms which occurred during labor before the operation. Without doubt, one of the most important points to be established is the size of the fœtal head, and the capacity of the woman's pelvis. When the disproportion between these structures is excessive, the danger of rupture is not so great as under other circumstances where the disproportion is slight, but not great enough to prevent the entrance of the head into the excavation, pushing a cap of the uterine tissue in front of it, and pinching the latter between the head and bony parts of the woman. It is then that, after a time, the uterine walls become weak and give way during a contraction.

2. *Case of Tubal Pregnancy.* JOHN COOKE, M.D. (*West Virginia Med. Student*, April, 1876.)

The subject, aged 35 years, multipara; last pregnancy thirteen years before. Her catamenia did not appear in June, though she had no morning sickness or other evidences of pregnancy. July 6, while ironing, she felt a sharp pain in the right iliac region, which soon subsided. During the evening of same day, while walking, she was again seized with pain in the region of right ovary, soon extending over the lower region of the abdomen. When first seen she was suffering from great pain in the abdomen, and physical prostration. Next morning she was free from pain; increased tenderness over lower abdominal region; countenance anxious and pinched. In the evening, pain had returned; restless; extremities cold; pulse small and rapid. The second morning, general condition not changed; there were now evidences of peritonitis. Tubal pregnancy, with rupture of the sac, was spoken of but not agreed upon by the counsel.

Patient died sixty hours after the first attack. At the autopsy, fully six pounds of coagulated blood was removed from the abdominal cavity; bowels distended with gas but not inflamed. The right Fallopian tube was much enlarged and inflamed; upon its anterior inferior surface, about one inch from the fimbriated extremity, there was a rent, one and one-half inches in length, through which a six-weeks fœtus had escaped.

3. *Eclampsia from Mastitis.* Dr. PANTHEL, Ems. (*The Clinic*, April 8, 1876.)

The woman, aged 25 years, had never had convulsions. About a year after marriage she gave birth to her first child after an easy labor. There was nothing abnormal about the puerperal week, except that she had scarcely a trace of milk, though her breasts were well developed. The child died at the end of four weeks. The woman had perfectly recovered, and was attending to her household duties. Six weeks after her confinement, she was suddenly seized with convulsions. During the visit of her attendant she was again attacked while speaking; her head was extended, eyes fixed, and her whole body was thrown into tonic and clonic spasms; there was complete loss of consciousness. The attack lasted about a quarter of an hour. Upon recovering from this attack, she complained of unpleasant sensations in her head and right breast; this organ was hard and inflamed. The attacks recurred at varying intervals for the space of four days and nights; at the end of this time, the mammary abscess had matured. The first convulsion was on Tuesday, and on Saturday morning the abscess was opened; the woman now rapidly recovered. The convulsions resembled those seen in the puerperal woman.

4. *Conception forty hours after Abortion.* SPARKMAN. (*Charleston Med. Jour. and Rev.*, April.)

The subject was about twenty-six years old; married. She had had four children and three abortions. The writer speaks of her as being

"of ardent temperament, very domestic, and devotedly attached to her husband." About four hours before her attendant's visit, she aborted a two-months fœtus. Twenty-seven hours afterward, she reported herself as follows: "I have been perfectly *dry* since morning and never felt better in my life." Her husband left her the following morning, and was absent for twenty-two days. Three weeks subsequent to the abortion, she consulted her attendant concerning a nausea which she had suffered for three weeks; she feared she was pregnant; and so she proved to be, for her attendant delivered her of a ten-pound baby two hundred and seventy-eight days from the time of the first visit after the abortion. The woman and the husband admitted sexual intercourse on two occasions during the two days following the abortion.

5. *Twins with different Birthdays.* Kollock. (*Charleston Med. Jour. and Rev.*, April.)

Dr. Kollock writes to the above Journal "that a negro woman was delivered, Oct. 3, at 5 P. M., of a living child, and he was called to her on the 4th, at 6.30 A.M., to deliver her of another, which was born fourteen hours after the first."

